# Management of Pediatric and Adolescent Type 2 Diabetes

**DOI:** 10.1155/2013/972034

**Published:** 2013-10-23

**Authors:** M. Constantine Samaan

**Affiliations:** Department of Pediatrics, Division of Pediatric Endocrinology, McMaster Children's Hospital, McMaster University, 1280 Main Street West, Room 3A-57, Hamilton, ON, Canada L8S 4K1

## Abstract

Type 2 diabetes (T2D) was an adult disease until recently, but the rising rates of obesity around the world have resulted in a younger age at presentation. Children who have T2D have several comorbidities and complications reminiscent of adult diabetes, but these are appearing in teens instead of midlife. In this review, we discuss the clinical presentation and management options for youth with T2D. We discuss the elements of lifestyle intervention programs and allude to pharmacotherapeutic options used in the treatment of T2D youth. We also discuss comorbidities and complications seen in T2D in children and adolescents.

## 1. Introduction

Type 2 diabetes (T2D) is increasing in youth, driven by the obesity epidemic that is affecting millions of children around the world [1]. T2D is a serious disorder with multiple complications that appear early in the course of the disease. Since T2D in young people has been only recognized over the past few years, knowledge of its natural history is lacking, and there are only few studies examining treatments beyond metformin and insulin. Even in case of the latter two medications, randomized controlled trials are very few, and knowledge is still accumulating in this field.

One study whose results were published about the role of different treatment modalities in T2D is the treatment options for type 2 diabetes in youth (TODAY) study [2]. This was a large, longitudinal, randomized, multicenter study that recruited 699 children and adolescents with an age range of 10–17 years and female to male ratio of 2 : 1. These patients were randomized to three treatment groups that included metformin alone or in combination with lifestyle intervention (LSI) or rosiglitazone. The mean time since diagnosis of T2D was 7.8 months and HbA1c less than 8% on enrollment. 

The primary outcomes, defined as failure to maintain HbA1c less than 8% over 6 months or metabolic decompensation requiring insulin therapy at diagnosis or restarting after stopping insulin within 3 months, occurred in 51.7%, 46.6%, and 38.6% in the above groups, respectively [2]. Metformin alone was no different from metformin plus LSI in improving metabolic outcomes, and higher failure rates in black participants were noted. Combination therapy of metformin plus rosiglitazone offered better success rates especially in girls but was associated with more weight gain. 

Despite intensive LSI, the rates of clinically important weight loss (7% or more) were achieved in only 24.3% in the metformin group, 31.2% in the metformin plus LSI groups, and in only 16.7% in the metformin plus rosiglitazone group [2].

This study revealed that, even with intensive LSI and pharmacotherapy, a significant number of T2D patients fail to achieve adequate glycemic control. In addition, the treatment options available to youth with T2D are limited when compared to adults, with insulin and metformin being the main agents used [3]. Furthermore, rosiglitazone has been associated with unfavorable cardiac effects that lead to limited use in adult patients with T2D, although this has been recently questioned [4], but this limits its use in youth at this point. 

In this review, we will discuss the diagnosis and treatment of T2D in teens in view of the results of TODAY study.

## 2. Clinical Presentation of T2D in Children and Adolescents

The average age of T2D diagnosis in youth is around 13.5 years, with female predominance. This age of presentation is likely to be related to a time of puberty-mediated insulin resistance in combination with increased weight [5]. 

The clinical presentation can be diverse. T2D can be detected while screening asymptomatic youth because of belonging to a high-risk population [6]. These risk factors include being overweight (BMI ≥ 85th percentile) or obese (BMI ≥ 95th percentile), family history in a first or second degree relative of T2D, being from certain ethnic groups known to have higher risk of T2D (Aboriginal, South Asian, Asian, African, and Hispanic), and history of in-utero exposure to obesity or hyperglycemia [7–9]. 

Additional risk factors that warrant screening for T2D include the presence of insulin resistance, for example, Acanthosis nigricans, dyslipidemia and hypertension, polycystic ovarian syndrome (PCOS), non-alcoholic fatty liver disease (NAFLD), and history of antipsychotic medication use [6–8]. The cost-benefit analysis for having a screening program for the general population is unjustified because of the low yield noted on several studies [10–14].

Screening in high-risk groups is recommended to start at the age of 10 years or when puberty starts if it is sooner than that, using fasting plasma glucose every 2 years. Oral glucose tolerance test can also be used but has poor reproducibility and is more expensive [6, 7]. 

Some children and adolescents present with diabetes-related symptoms including polyuria, polydipsia, tiredness, blurred vision, vaginal moniliasis, and weight loss [6]. They may also present with acute metabolic decompensation including ketosis, diabetic ketoacidosis, and hyperglycemic hyperosmolar nonketotic state [15]. 

## 3. Laboratory Diagnosis of T2D in Children and Adolescents

The laboratory diagnosis of T2D in children uses the blood glucose cut-offs that are identical to adults and involves measuring fasting or random plasma glucose or a formal oral glucose tolerance test [7, 16, 17]. HbA1c is not recommended in the pediatric age group as a diagnostic test as is the case in adults but is used for follow-up in established T2D to determine glycemic control [7].

One area of difficulty in confirming the diagnosis of T2D is its overlapping picture with type 1 diabetes (T1D) and less so with familial diabetes (MODY). Because the general population is becoming overweight and many families do have a history of diabetes and as up to 33% of T2D youth have ketonuria/ketonemia at diagnosis, the distinction between T1D and T2D is challenging in this subset of patients. Pancreatic autoantibody testing is positive in 10–75% of T2D patients, and this may indicate that these patients with “autoimmune type 2 diabetes” are in fact overweight type 1 diabetes patients [18–21], as clamp studies have revealed that those with antibodies are more insulin resistant and have reduced insulin production capacity [22]. However, this is not fully clarified as some children who phenotypically seem to have bona fide T2D have antibodies [23], and one additional possibility is that islet injury due to glucotoxicity and lipotoxicity exposes cellular antigens that lead to an immune response as a secondary phenomenon. 

## 4. Management of T2D in Children and Adolescents

The current management plans for T2D involve lifestyle intervention and pharmacotherapy. 

The treatment of T2D requires a family-focused plan delivered by a multidisciplinary team with expertise in dealing with T2D in youth and taking into account the significant differences between T2D and T1D. Success in treating T2D requires addressing the main mechanisms that lead to its development, including insulin resistance and *β*-cell failure. The evidence for benefit of bariatric surgery in teens with T2D is currently limited [24, 25], and there are no data to justify its use out of research settings, but this is a fast-moving field.

The multidisciplinary team includes a combination of physicians, diabetes nurse educators, dietitians, physical activity specialists, social workers, psychologists, and behavioral therapists and may also require the involvement of additional medical subspecialties to address its comorbidities and complications [8]. 

The management plan should be intensive with frequent contacts with family and teen and personalized to the individual patient taking into account the family's financial resources and being receptive and respectful of ethnic and cultural attributes of the family [26]. Engaging the patient and family early and frequently is critical to minimize attrition, which is a common problem in this population [27]. 

The goals of T2D management include

- achieving and maintaining glycemic control,
- weight maintenance or weight loss if possible,
- acquisition of healthy lifestyle habits and skillsets,
- management of comorbidities,
- prevention of complications.

The above goals can be met with establishing an education plan and the introduction of lifestyle intervention program (LSI), along with the use of pharmacotherapy. It is important to note that there are no evidence-based guidelines for glycemic goals for teens with T2D, and HbA1c targets include 7% or less for American Diabetes Association, Canadian Diabetes Association, and International Society of Pediatric and Adolescent Diabetes [7, 16, 28] and is set at 6.5% for International Diabetes Federation [29].

Pharmacotherapy and, perhaps in the future, surgery need to consider that many patients are still growing and developing, and the long-term effects of these interventions in teens are unknown. However, the complications of poorly managed diabetes already documented justify the current approach.

## 5. Lifestyle Intervention Program (LSI)

One of the most important steps in T2D management is the implementation of LSI for the T2D teen and their family. The elements of this program should highlight the significance of the diagnosis of T2D in this age group and should involve advice about behavioral changes including modifying nutrition, increasing physical activity, reducing screen time, and maintaining good sleep hygiene measures [8, 16, 30, 31]. 

LSI works best when the whole family engages in learning about supporting the teen, and these recommendations may be helpful for other family members who may suffer from obesity and T2D. Parents and caregivers need to be informed of the importance of modeling of healthy behaviors as a way to encourage children to acquire healthy habits. They also need to maintain positive reinforcement of success and avoid penalizing failures [8, 31]. The key is to build collaborative relationships and avoid combative interactions when it comes to managing T2D.

### 5.1. Nutrition

Regarding nutrition, focus should be on formulating a nutrition plan that involves food composition and eating behaviors that drive excess food intake [31]. Recommendations regarding controlling portion size and setting up regular meal and snack times are important. Equally important is eating meals as a family and elimination of distractions during meal times including TV, computers, or other disturbances to slow down eating and improve social interactions. Other recommendations include avoiding snacking especially while watching TV, using the computer, and late at night [31–33]. 

In addition, the elimination of sugary drinks and foods with high fat or caloric content is also critical to reducing caloric intake to promote weight maintenance or weight loss. Parents, caregivers, and teens need to be taught how to read food labels to understand the nutritional value of consumed foods and to emphasize the importance of consuming less fat including saturated fatty acids, increasing fiber intake, and reducing sugar intake and eating out [31, 32, 34, 35]. 

### 5.2. Physical Activity

Reducing sedentary time and improving physical activity and fitness levels are important goals of LSI for the teen and family. Physical activity increases energy expenditure, improves insulin sensitivity, and has several benefits beyond diabetes including reducing stress levels and mental health problems. The current recommendations for children's physical activity include 60 minutes per day of moderate-vigorous activity levels that can be done at the same time or broken into sessions, and providing a written exercise script may improve compliance [36].

In some T2D patients, vigorous activity may include using stairs at home and school, walking or biking to and from school, and walking to the local stores. It may also involve the participation in structured sporting activities which the teens find enjoyable and are willing to commit to doing [8, 37]. 

### 5.3. Screen Time

Increased screen time is associated with increased sedentary time and obesity. While there are no specific guidelines to address screen time use in T2D, the recommendations for prevention of childhood obesity by the American Academy of Pediatrics recommend limiting screen time to two hours per day excluding use for academic purposes or for work [38] and seem reasonable to follow in T2D.

Sustaining LSI is a challenge in the T2D population, and in one study only 17% of patients have lowered their BMI over 1 year of LSI implementation, and 23% were off medications over 2 years [27]. 

LSI is essential not only to manage T2D per se but more so to deal with its associations including fatty liver disease and to modulate future cardiovascular risk. 

## 6. Pharmacotherapy

While LSI is important to provide the basis for acquiring healthy behaviors in T2D, the success rates of maintaining glycemic targets based on LSI alone are low [31], and starting pharmacotherapy at diagnosis is appropriate.

### 6.1. Metformin

Metformin is the first line therapy for youth with T2D [31]. It is a biguanide that lowers blood glucose levels via several mechanisms including

- reducing hepatic glucose output by inhibiting gluconeogenesis [39, 40],
- increasing insulin-stimulated glucose uptake in muscle and adipose tissue [41, 42],
- inhibiting inflammation in cells by inhibiting the NF*κ*B pathway which, when active, interferes with insulin signaling [43],
- increasing fatty acid oxidation in muscle and inhibiting fatty acid synthesis in fat and liver by upregulating AMPK activity [44–46],
- enhancing the secretion of GLP-1 from gut [47].

A randomized clinical trial using metformin for 16 weeks in children showed significant improvement of HbA1c [48], but was used for few years in TODAY study with no major adverse effects. Metformin has an initial anorexic effect and may result in modest weight loss. It lowers HbA1c by 1-2% and is to be taken with food to minimize its gastrointestinal side effects including nausea, vomiting, diarrhea, and abdominal pain. 

There are slow release preparations (e.g., Glucophage XR, Glumetza) that have less gastrointestinal side effects and are taken once daily, which may improve compliance, and pediatric trials are ongoing to evaluate their efficacy. Importantly, metformin use is rarely associated with hypoglycemia [8, 49].

Metformin needs to be avoided in renal impairment, liver disease, cardiac or respiratory insufficiency, and when using radiographic contrast media. Metformin may also improve ovarian function, and this increases the chances of pregnancy. 

The usual maintenance dose is 1000 mg twice daily, with a starting dose of 250–500 mg, to be increased every few days to reach full dose within 3-4 weeks. If side effects develop, the patient can be reassured that these are transient and to continue titration with lower dose increments [8].

### 6.2. Insulin

Insulin reduces islet glucotoxicity and has a paradoxical effect on improving insulin sensitivity in the context of insulin resistance. Insulin is used at presentation if the patient is hyperglycemic (blood glucose > 11.1 mmol/L), ketotic, or ketoacidotic or if the HbA1c is ≥9%. 

The goal of insulin therapy is to reverse the acute metabolic decompensation noted in some patients at presentation and may be used for few weeks at diagnosis along with metformin and then is withdrawn gradually [50]. In some cases, it is not possible to come off insulin, and, in one study, only 28% of those started on insulin managed to wean down treatment, and, of those who successfully came off insulin, the majority restarted therapy due to deterioration of metabolic control [51]. In addition, insulin is usually needed few years after the initial diagnosis on a maintenance basis due to *β*-cell failure.

There are few pediatric data on the insulin regimens used, but they all seem to have equal efficacy. In one study, insulin premix 30/70 was used twice daily for 8.7 ± 4.3 weeks in 18 aboriginal adolescents at a dose of 0.5 units/kg/day. This study showed a significant decline in HbA1c that persisted for 12 months after insulin cessation [50]. There were no significant hypoglycemic episodes or BMI changes.

In addition, peakless insulin analogs including insulin Glargine are effective in adult studies as a single daily dose when compared to insulin lispro multiple daily injections in T2D patients who are on added oral hypoglycemic agents [52]. This may be a useful strategy, as adolescents are likely to comply with single daily injection regimens [53]. The Glargine dose for adolescents who fail oral therapy and LSI is 0.3–0.4 units/kg/day [16]. 

Insulin may be the only prescribed agent in adolescents with T2D in several institutions [49, 51]. The main side effects of insulin include weight gain and hypoglycemia [54]. 

### 6.3. Other Medications

There are limited data on the use of additional medications to treat T2D in youth compared to adults. There are ongoing studies to evaluate their role alone or in combination to treat youth. 

#### 6.3.1. Sulfonylureas (SU)

Sulfonylureas are insulin secretagogues and therefore are only effective if residual pancreatic insulin secretion is present. They bind to the sulfonylurea receptor on the *β*-cell; this results in closure of the K_ATP_ channel and depolarization of the cell membrane and calcium influx through the calcium channels, which results in insulin release. 

These drugs are not widely used in the pediatric population. In one study, a comparison between glimepiride (1–8 mg once daily dose) and metformin for 24 weeks showed equivalent HbA1c drop but more significant weight gain [55]. The other side effects of SU involve hypoglycemia, nausea, vomiting, diarrhea, and constipation. 

#### 6.3.2. Thiazolidinediones (TZD)

Rosiglitazone and pioglitazone are the only remaining drugs from TZD family in clinical use, and rosiglitazone was used in the TODAY study.

Rosiglitazone binds to peroxisome proliferator-activated receptor gamma (PPAR-*γ*) in metabolic cells. This is a transcription factor and master regulator of fat and carbohydrate metabolism and is an insulin sensitizer. In adults, rosiglitazone reduces HbA1c by 0.5–1.3% [56].

Recently, an FDA panel voted to ease the restrictions for rosiglitazone use in adults with T2D, as previous reports that suggested a possible association with adverse cardiovascular outcomes were felt to be exaggerated [4]. This may result in increased use in the adult population and may offer further basis for its use in children. However, progress will be slow until more evidence becomes available.

#### 6.3.3. Incretin Mimetics

This class of drugs includes glucagon-like peptide-1 (GLP-1) receptor agonist, which is a peptide secreted by the L cells of the small intestine in response to food, and has a half-life of 2 minutes [57, 58]. It enhances insulin secretion in response to glucose, suppresses glucagon production, delays gastric emptying, prolongs satiety, and reduces HbA1c and weight. It is given subcutaneously twice daily, which may limit compliance in teens. Some of its side effects include nausea, vomiting, diarrhea, dyspepsia, and headache [59–61]. Pediatric studies are ongoing to validate its use in T2D teens.

#### 6.3.4. DPP-IV Inhibitors

These agents inhibit dipeptidyl peptidase- (DPP-) IV, the enzyme that degrades incretin hormones. DPP-IV inhibitors thus increase the activity of endogenous glucagon-like peptide-1 (GLP-1). They do not affect gastric emptying, satiety, or weight. They are given once daily orally with metformin, and there are no pediatric data to evaluate their role in T2D [62].

#### 6.3.5. Amylin

This is a peptide released with insulin by the *β*-cells at a ratio of 1 : 100. It reduces glucagon production, slows gastric emptying, and reduces food intake. It is given to patients who are on insulin and causes a mild reduction in HbA1c and mild weight loss but is associated with nausea and hypoglycemia that requires the reduction of insulin dosing by as much as 50% [63]. There are no data on its use in T2D youth.

#### 6.3.6. Alpha Glucosidase Inhibitors

This class of drugs delays the absorption of carbohydrates by inhibiting the breakdown of oligosaccharides in the small intestine. It can reduce HbA1c by 0.5–1%, and in the year 2000 about 4% of T2D adolescents were using this drug. It needs to be taken before every meal, and flatulence is a side effect [8, 49]. Both of these reasons make them less desirable for the T2D teen.

### 6.4. Bariatric Surgery

In adults, bariatric surgery in T2D results in the remission of T2D and discontinuation of medications [64, 65]. In the only pediatric study published so far, 11 adolescents with T2D who underwent Roux-en-Y gastric bypass were compared to those who were medically managed; the former group had around 34% reduction in BMI and improved control of hypertension and dyslipidemia. In 10 patients from the surgical group, T2D disappeared and they did not require pharmacotherapy. As this is a new procedure in T2D teens, longitudinal studies are needed to validate its feasibility [25].

## 7. Comorbidities of T2D

It is important to note that T2D is associated with other comorbidities that are related to insulin resistance, and some of these comorbidities are present at diagnosis. T2D is a more aggressive disease than T1D, with complications occurring early in the course of the disease. Over the past few years, the term “metabolic syndrome” emerged as a unifying description of insulin resistance-related conditions and includes abdominal obesity, dysglycemia, dyslipidemia, and hypertension. Insulin resistance is also associated with hyperandrogenism and polycystic ovarian syndrome (PCOS) and non-alcoholic fatty liver disease (NAFLD).

### 7.1. Proteinuria

Microalbuminuria (≥2.5 mg/mmol) or macroalbuminuria is far more common in T2D when compared to the dominant form of diabetes in children, that is, type 1 diabetes (T1D). In the search for diabetes study, microalbuminuria was present in 22.2% of T2D versus 9.2% in T1D patients [66]. In another study from Canada, 14.2% of T2D subjects had proteinuria. The rate of progression of microalbuminuria is also fast in T2D [67].

Screening for proteinuria should start at diagnosis and annually afterwards. It should be confirmed on 2-3 samples [68]. Screening can be done initially with a random or early morning albumin : creatinine ratio (ACR), and, if the result is abnormal, this should be confirmed with another early morning ACR 4 weeks later. If the two results are abnormal, a timed overnight urine collection for ACR should be done. The diagnosis is made by repeated samples over 3-4 months, and, if persistent over 6–12 months, then a referral to nephrology service for further evaluation is warranted [7, 69, 70].

### 7.2. Hypertension

Hypertension (BP ≥ 95th percentile for age, sex, and height and confirmed on two readings) is present in 20–30% at initial presentation. BP should be checked at diagnosis and with every encounter afterwards.

When hypertension is associated with proteinuria, it can progress to end-stage renal disease (ESRD) and requires aggressive treatment [71]. Hypertension may account for 35–75% of micro- and macrovascular problems in T2D [30].

Treatment involves using angiotensin converting enzyme inhibitors (ACEI) or angiotensin receptor blockers (ARB) [16].

### 7.3. Dyslipidemia

This is present in 50–65% of T2D teens with established T2D, with hypertriglyceridemia and low high density lipoprotein (HDL) being the most common abnormalities seen [30].

In children who have family history of dyslipidemia or early cardiovascular disease and after 3–6 months of unsuccessful LSI, statin therapy is recommended. The goals of therapy are to maintain LDL below 2.6 mmol/L, triglycerides below 1.7 mmol/L, and HDL above 0.9 mmol/L [30, 33, 72, 73]. Statins are the first line of therapy in these patients [33].

### 7.4. Non-Alcoholic Fatty Liver Disease (NAFLD)

Deposition of fat in the liver is a common association with T2D, with 22.2% of children having NAFLD at diagnosis [74]. Fatty liver is related to increased visceral fat mass and insulin resistance and components of the metabolic syndrome and is twice as common in boys [75, 76]. This condition is first suspected with elevated transaminases (×3 times normal levels), and the diagnosis is confirmed on a liver ultrasound. The treatment for NAFLD is LSI including weight loss and exercise [77]. 

### 7.5. Polycystic Ovary Syndrome (PCOS)

PCOS is seen in obese and T2D women, including adolescents [78, 79]. The diagnosis criteria are not fully formalized in teens because several features of PCOS are seen during the course of normal puberty. However, emphasis on using more rigorous criteria for teen PCOS diagnosis has gained more support, including the recently revised Rotterdam criteria and others; these involve having oligo- or anovulation or primary amenorrhea at 16 years of age, clinical and biochemical hyperandrogenism, and ovarian volume of ≥10 cm^3^ on ultrasound (need 3 of 3) [80–82]. 

Patients with PCOS require an oral glucose tolerance test as there is a higher rate of dysglycemia associated with the diagnosis [83]. Some of the features of PCOS result from excess insulin actions including increasing ovarian testosterone production and reducing hepatic sex hormone-binding globulin production [84]. 

The treatment of PCOS involves lifestyle intervention; in addition, combined oral contraceptive pills, antiandrogens (e.g., spironolactone), and insulin sensitizers including metformin all play a role in different patients [83, 85].

### 7.6. Obstructive Sleep Apnea (OSA)

There is evidence that OSA is associated with the metabolic syndrome [86]. In adults, OSA patients have higher rates of NAFLD [87], and patients with PCOS have higher rates of obstructive sleep apnea. The diagnosis is suspected clinically and confirmed on sleep studies. In some cases, OSA is treated with CPAP.

## 8. Complications of T2D

Youth with T2D have a more aggressive disease than adult T2D and pediatric T1D, with complications noted early in the course of the disease. 

The incidence of nephropathy in Pima Indians, a population with the highest rate of T2D in the world, is the same across different age groups, and retinopathy was more common when T2D is diagnosed at an older age [88].

In addition, over the course of the TODAY study (mean follow-up 3.9 years), one-third of patients were hypertensive, one in six had proteinuria, and none had retinopathy [2]. 

In another study, retinopathy was noted in 4% of T2D, proteinuria and hypertension in 36%. In the latter study, peripheral and autonomic neuropathy occurred at 21 and 57% of T2D teens, respectively [89]. It has also been shown that renal disease and retinopathy may occur early in the course of the disease, and, by early adulthood, many of these complications were already causing morbidities [90]. This indicates the need for aggressive screening for complication in T2D youth at diagnosis and regularly afterwards, and collaboration with nephrology, ophthalmology, and neurology services is a must.

## 9. Conclusions

T2D in youth is an emerging disease and is more aggressive than T1D, the more common diabetes form in children. The complications seen in T2D are extensive, and many patients have adverse health problems related to diabetes at diagnosis. Due to its novelty, this disease is not fully understood, and treatment options beyond metformin and insulin are limited. 

Over the coming years, randomized controlled trials are needed to test new and old treatments including combination therapies to define the best treatment options for these patients. Further understanding of the natural history of disease-related comorbidities is required to determine optimal screening times and frequencies and define appropriate interventions to limit their effects. 

Unfortunately, this will take time, which is something these youth do not have as they battle this disease. There is a need to further define the mechanisms that cause T2D and its comorbidities and complications, so that targeted therapies may be implemented. 
